# Progress in Surgery

**Published:** 1896-12-05

**Authors:** 


### Progress in Surgery.

### SURGERY OF THE PERITONEUM, &c.

**Peritoneal Adhesions** are frequently the cause of much abdominal pain, and Bouquet de Jolinière[1] has shown how various diseases may be simulated by symptoms dependent on chronic peritoneal adhesions. Adhesions of the stomach cause pain, indigestion, and vomiting. Pain in the epigastrium is a constant feature, coming on before or after meals, and often increasing in intensity until the patient vomits. It is of a stabbing or dragging character, and is generally absent when the stomach is empty, and after an attack of vomiting. The author points out how these symptoms may be mistaken for gastric ulcer. Intestinal adhesions also cause pain of a variable character; and adhesions in the neighbourhood of the gall bladder cause pain closely resembling biliary colic. Operative treatment is the most promising, and, on the whole, the prognosis is good. Krogius[2] records a severe operation which he performed for the relief of serious and very painful symptoms caused by adhesions of the stomach to the abdominal wall after the healing of a gastric ulcer. The patient, a woman, aged fifty-one, had the usual symptoms, but of a very severe type, and she was much emaciated. In the epigastrium, near the ribs on the left side, a firm, uneven tumour, about two and a-half inches long, could be felt in the abdominal parietes, but apparently connected with the stomach. A median incision was made from the ensiform cartilage to the umbilicus, and the swelling was found to be due to a dense cicatricial deposit, which was so closely adherent to the stomach that it was found necessary to excise the involved portion of the anterior stomach wall. The operation lasted over two hours, but the patient ultimately recovered, and, when seen six months after operation, was found to be still free from pain.

**Tuberculous Peritonitis** should, says Abbé,[3] be looked at from the standpoint of the bacillus if we want to understand the multiform appearances of the disease. A sudden attack of tuberculosis of the peritoneal cavity may cause symptoms as acute as those of peritonitis from other causes. Slower outbreaks may not induce ascites and wasting for three or four weeks or as many months. In other cases, possibly due to the penetration of bacilli through lymphatics communicating between the mucous and serous coats of the intestine, a dry or adhesive variety follows, in which we get hectic and rapid wasting. Or, again, we may get an outpouring of thick lymph and flocculent serum rapidly becoming purulent, and producing thickened omentum, matted intestines, and encysted collections of pus. Thus the life history and products of the bacillus are represented by the various phases of the disease. Operative treatment of the peritonitis has often been followed by a general cure, even when the pleura or other parts have been affected. Laparotomy is by far the best method of treatment. The peritoneum should be irrigated with warm salt solution. Camphor-naphthol application has been advised by Rendu for very advanced cases. Abbé thinks the best theory for accounting for the many wonderful cures of this disease after abdominal section is that based on the life history of tubercle bacillus, and the capacity of the animal economy for suppressing the growth of the organism by encapsulating it, and subsequently removing it by absorption. The presence of ascitic fluid acts almost as a culture medium, but when the peritoneum has been roused into activity by evacuation of the fluid, a reactionary inflammation is set up, producing a cell hyperplasia resulting in favourable cases in the death of the intruding organism.

**Acute General Peritonitis** is almost invariably fatal when left alone, whatever the cause may be, and operation only meets with a qualified amount of success. Heaton,[4] however, records two cases in which recovery followed after simple incision and drainage of the peritoneal cavity. In one case the cause of the peritonitis was not found; in the other, it appears to have been of an acute tuberculous nature, which Abbé[5] has pointed out as being sometimes as severe as any form of peritonitis.

**Weak Scars and Hernia following Laparotomy** are affected by two conditions, according to Barker[6]—firstly, the presence of suppuration, in which the repair of the abdominal wall is effected by the production of a large amount of imperfectly formed, badly vascularised and weak fibrous tissue; and secondly, the healing of the wound by first intention, in which we get a minimum of fibrous tissue, and that material is highly organised and very strong. It is therefore of the utmost import- to prevent suppuration; or, if that be impossible, to limit it in time and extent by proper drainage. It is by no means always necessary to drain all cases of intra-abdominal suppuration, as the peritoneum is capable of absorbing a certain amount of pus. Another important point in the strength of the scar is the inclusion of muscular fibres in the stitches, and our incisions should by preference be made through muscle rather than through an aponeurosis. The muscular fibres themselves should not be cut, but merely separated, so that when they resume their old place, the abdominal wall is left practically as solid as before. Barker applies these principles to operations on ventral hernia. The edges of the wound are freshened, and the weak tissue cut out. The peritoneum is left to look after itself. Silver sutures are passed very deeply, and far from the edge of the recti on each side, and include all the structures of the abdominal wall except the skin and peritoneum. The stitches are about a quarter of an inch apart, and are left in permanently. As the wires are twisted up, the edges of the recti are inverted so as to bring the outer surface of the muscles back to back, and thus a large surface between the edges of the rectus on both sides is obtained.

**The Sub-peritoneal Tissue** is of much surgical importance. Anderson[7] draws attention to its anatomical relations, and points out that in consequence of its close contact with the pelvic wall and the vertebræ it is liable to be affected by all diseases of these structures. It is also necessarily implicated by affections of the peritoneum, and from its structure is liable to certain new growths. The author groups the varieties of inflammation of the sub-peritoneal tissue regionally and ætiologically. The regional group includes (1) the retro-peritoneal, (2) the sub-phrenic, (3) the perinephric, (4) the iliac, (5) the pelvic, and (6) the pre-vesical. The etiological classification is more important, and includes—(1) The idiopathic, (2) the tuberculous, (3) the syphilitic, (4) the metastic, (5) the traumatic, and (6) the consecutive forms. This last is the most extensive. A sub-peritoneal abscess may arise in the tissue itself, or in structures around it, or even in structures outside the abdomen, as after varicocele. Such abscesses always tend to reach the surface, and rarely open into a viscus as intra-abdominal abscesses do. The new growths of this tissue are little known. Lipomata grow rapidly and to an enormous size, and surround the abdominal vessels. A hernial variety of lipoma is most commonly met with in the inguinal canal, and may be productive of a true hernia by drawing down a pouch of peritoneum. The fibromata may reach a large size, and, as in the case of the lipomata, are generally too complicated in their relations to admit of surgical treatment. Sarcomata are comparatively common, but are utterly beyond treatment. Hæmorrhages, as from a ruptured aneurism, and lymph exudations may occur; and other matters, such as bile and fæces, may be extravasated into the tissue. Gaseous infiltrations, of pulmonary or intestinal origin, may occur. An atrophic form of disease may be the cause of movable kidney, especially in women. Morton[8] and MacDougall[9] refer to cases of sarcoma of this tissue; and Ogston,[10] MacDougall,[11] and Laurenstein,[12] reports cases of sub-peritoneal abscess, lipoma, and myoma.

**Foreign Bodies in the Peritoneal Cavity,** from the accidental inclusion of sponges, forceps, &c., after operation, have been the cause of death in many instances. MacLaren[13] reports a case of ovariectomy and hysteropexy in which a gauze sponge was left in the peritoneal cavity, but expelled from the rectum ten days after; and another case on which he operated for pain and tumour in the right loin two years after the performance of hysterectomy, and the tumour was found to be caused by a pair of artery forceps embedded in ulcerated intestine and enclosed in omentum. Both patients recovered. The author also refers to ten cases reported by Wilson, in which the leaving of a sponge after cœliotomy had been followed by a fatal peritonitis. Since the days of a more strict attention to antiseptics the results have not been so fatal, seven cases being recorded with only one death, but two others would undoubtedly have died if surgical assistance had not been afforded. Coe, in a personal letter to Elsner,[14] says: "I have removed four sponges, post-mortem, from the cavity, which had been left by eminent operators."

**Thirst after Cœliotomy** is a most distressing symptom, and Humiston[15] maintains that the method which he previously[16] advocated is still found successful in preventing thirst, and increasing the amount of urine passed after abdominal section. His method consists in ordering the patient to drink a pint of hot water an hour before meals and on retiring, for three days prior to the operation. The last pint is to be taken three hours before the time of operation. In this way, he states, that the loss of fluid caused by the free catharsis usually ordered before an abdominal section is restored, and that the first thirty-six hours after operation will therefore be passed in comparative comfort, with a moist tongue, an active secretion of urine, and no thirst.

**Collapse after Cœliotomy,** says Braithwaite,[17] quoting Horrocks, can usually be successfully treated by transfusion, even although the cause of the collapse is not hæmorrhage, and although the collapse may not occur for some hours after operation. He quotes a case of collapse after myomectomy in which this treatment, combined with the administration of oxygen (for the patient was suffering severely from ether bronchitis), appears to have saved the patient's life.

[1] Brit. Med. Journ., June 13th, 1896, and Journ. de Med., April 25th, 1896. [2] Centralbl. for Chir., No. 22, 1896, and Brit. Med. Journ., June 15th, 1896. [3] New York Med. Rec., June 13th, 1896. [4] Practitioner, Aug., 1896. [5] Op. Cit. [6] Clinic. Journ., July 22nd, 1896. [7] Lancet, Aug. 15th, 1896. [8] Ibidem. [9] Ibidem. [10] Ibidem. [11] Ibidem. [12] Ibidem. [13] Annals of Surg., Sept., 1896. [14] Amer. Gynæ. and Obstet. Journ., Mar., 1895. [15] Columb. Med. Journ., Aug. 18th, 1896. [16] Amer. Journ. of Obstet., July, 1895. [17] Brit. Med. Journ., Oct. 10th, 1896.

